# Characterization of CD147, CA9, and CD70 as Tumor-Specific Markers on Extracellular Vesicles in Clear Cell Renal Cell Carcinoma

**DOI:** 10.3390/diagnostics10121034

**Published:** 2020-12-02

**Authors:** Dirk Himbert, Philip Zeuschner, Hiresh Ayoubian, Joana Heinzelmann, Michael Stöckle, Kerstin Junker

**Affiliations:** 1 Department of Urology and Pediatric Urology, Saarland University, 66421 Homburg/Saar, Germany; dirk.himbert@uks.eu (D.H.); Philip.Zeuschner@uks.eu (P.Z.); hiresh.ayoubian@uks.eu (H.A.); joana.heinzelmann@uk-halle.de (J.H.); michael.stoeckle@uks.eu (M.S.); 2 Department of Ophthalmology, University Hospital Halle (Saale), Martin-Luther-University Halle-Wittenberg, 06108 Halle/Saale, Germany

**Keywords:** extracellular vesicles, tumor exosomes, ultracentrifugation, biomarkers, liquid biopsies, clear cell renal cell carcinoma, sucrose gradient

## Abstract

Extracellular vesicles (EVs) are secreted by healthy and tumor cells and are involved in cell–cell communication. Tumor-released EVs could represent a new class of biomarkers from liquid biopsies. The aim of this study was to identify tumor-specific EV markers in clear cell renal carcinoma (ccRCC) using cell lines and patient-derived tissue samples. EVs from ccRCC cell lines (786-O, RCC53, Caki1, and Caki2) and patient tissues were isolated via ultracentrifugation. EVs were characterized using transmission electron microscopy, nanoparticle tracking analysis, and Western blotting using exosome and putative tumor markers (epithelial cell adhesion molecule (EpCAM), carbonic anhydrase 9 (CA9), CD70, CD147). The tumor markers were verified using immunohistochemistry. CA9 was expressed in Caki2 cells and EVs, and CD147 was found in the cells and EVs of all tested ccRCC cell lines. In tumor tissues, we found an increased expression of CA9, CD70, and CD147 were increased in cell lysates and EV fractions compared to normal tissues. In contrast, EpCAM was heterogeneously expressed in tumor samples and positive in normal tissue. To conclude, we developed an effective technique to isolate EVs directly from human tissue samples with high purity and high concentration. In contrast to EpCAM, CA9, CD70, and CD147 could represent promising markers to identify tumor-specific EVs in ccRCC.

## 1. Introduction

Despite significant advances in diagnostics, there is a lack of adequate methods to detect cancers at early stages. However, early detection of cancer is one of the most promising approaches to improve therapy outcomes combined with an individual prognostic evaluation to enhance therapy selection [1,2,3,4,5,6].

In this regard, biomarkers are increasingly important in clinical management [7,8,9]. In particular, liquid biopsies from the blood and urine represent a promising source for biomarkers, as they reflect the complex spectrum of tumor cells and microenvironment alterations compared to tumor tissue biopsies. This minimally invasive approach represents another important advantage allowing for multiple analyses during the disease course [10,11,12]. Extracellular vesicles (EVs) represent a new putative class of biomarkers from liquid biopsies. These EVs with a diameter of 30–5000 nm are divided mainly into large (i.e., microvesicles) and small EVs (i.e., exosomes). In contrast to the larger microvesicles, exosomes with a characteristic size of 30–150 nm are actively secreted by almost all cells and play an important role in cell–cell communication and cell homeostasis. They are involved not only in physiological processes, but also in tumor development and progression. Their molecular properties at least partly reflect their parental cells, rendering them a promising source of biomarkers from liquid biopsies [13,14,15,16,17,18,19,20,21,22].

To date, diagnostic biomarkers for renal cell carcinoma (RCC) suitable for liquid biopsies are still lacking, although promising data have been published. The few studies on EVs are mainly focused on microRNAs (miRNAs) and proteins in serum and urine [23]. However, there is a low concordance between the reported exosomal markers.

In general, one has to consider that tumor-derived exosomes represent only a particular fraction of the exosomes in blood and urine in addition to those secreted by different organs and cells. Therefore, enrichment techniques for tumor exosomes via tumor- or cell-type specific markers might increase their diagnostic accuracy [23]. For example, the prostate-specific membrane antigen (PSMA) has already been used as a tumor-specific exosome marker in prostate cancer [24]. In addition, epithelial cell adhesion molecule (EpCAM) serves as another marker in prostate cancer, and also RCC patients [23,25]. However, EpCAM is an epithelial cell, but not a tumor-specific cell marker, and is even downregulated in aggressive dedifferentiated tumors including RCC [26]. Therefore, we selected carbonic anhydrase 9 (CA9), CD70, and CD147 as highly promising tumor specific markers on exosomes in RCC, since they have been shown to be upregulated during tumor development and progression in clear cell RCC [27,28,29,30].

CD70 interacts with CD27 and modulates the immune response by targeting antigen receptor and toll-like receptor stimulation on T-cells, B-cells, dendritic cells [31], and natural killer cells [32]. In addition to its general immune regulation function, it seems to be involved in clear cell RCC (ccRCC) tumorigenesis, as it was found to be upregulated compared to normal kidney tissue [33].

CA9 displays alterations in tumor metabolism and acid/base regulation, therefore enhancing tumor growth and metastasis. Acid/base homoeostasis in cancer cells is governed by the concerted interplay between carbonic anhydrases (CAs) and various transport proteins. By its catalytic function, CA9 is involved in cancer cell migration and invasion. Since CA9 is almost exclusively expressed in cancer cells, it might function as promising biomarker and therapeutic target [34]. The utility of CA9 to diagnose primary and metastatic ccRCC has already been demonstrated [35,36,37].

CD147, also called Basignin/extracellular matrix metalloproteinase inducer (EMMPRIN), is a glycoprotein with versatile functional properties: it is indispensable for the exchange of lactate between cells, acts as a cell adhesion molecule, and induces matrix metalloproteases (MMPs). Hence, it is involved in several tumor-supporting processes including matrix degeneration, tumor cell invasion, metastasis, and angiogenesis [38,39,40]. Upregulated CD147 leads to altered vascular endothelial growth factor (VEGF) and basic fibroblast growth factor (bFGF) expression, cell proliferation, and invasiveness of RCC cells [41].

In order to identify eligible tumor-specific exosomal markers in ccRCC, we examined the expression of these putative candidates in exosomes from RCC cell lines and primary patient-derived tumor tissues.

## 2. Materials and Methods

### 2.1. Patient Samples

Frozen and formalin-fixed paraffin-embedded (FFPE) tissue samples were obtained from patients with primary ccRCC undergoing surgery at our department. These samples were used for exosome isolation and immunochemistry. Written informed consent was obtained from all patients.

### 2.2. Cell Culture

Four different ccRCC cell lines (786-O, Caki1, Caki2, and RCC53) were cultivated in corresponding media (Dulbecco’s Modified Eagle’s Medium (DMEM), Roswell Park Memorial Institute-1640-medium (RPMI), DMEM/RPMI (1:1), Sigma-Aldrich, Darmstadt, Germany) containing 10% fetal calf serum (FCS, Sigma-Aldrich). The human breast cancer cell line MCF7 served as a positive control. Before EV isolation, the cells were incubated in medium with exosome-depleted FCS (ED-FCS) for 72 h. Then, the exosome-containing supernatant was removed and differentially centrifuged (2000 *g*/4 °C/20 min; 15,000 *g*/4 °C/30 min). The supernatants were further used for exosome isolation.

### 2.3. Exosome Isolation from Cell Culture Supernatants

Cell culture supernatants (160 mL/cell line) were centrifuged at 100,000 *g* and 4 °C for 90 min. The pellets were dissolved in 1 mL phosphate-buffered saline (PBS, Sigma-Aldrich) and centrifuged again at 100,000 *g* and 4 °C for 90 min. The pellet was dissolved in 50–100 µL PBS (for NTA) or lysis buffer (for TEM, with protease inhibitor mix). The protein concentration was determined via a bicinchoninic acid (BCA) assay (Pierce BCA Protein Assay Kit, Thermo Scientific, 58239 Schwerte, Germany). All samples were stored at −20 °C in lysis buffer/protease inhibitor, or at 4 °C in PBS.

### 2.4. Exosome Isolation from Tissue

We adapted and optimized isolation from renal tissues according to a published protocol for brain tissue [42] and for renal tissue from a master’s thesis [43].

**Figure 1:**
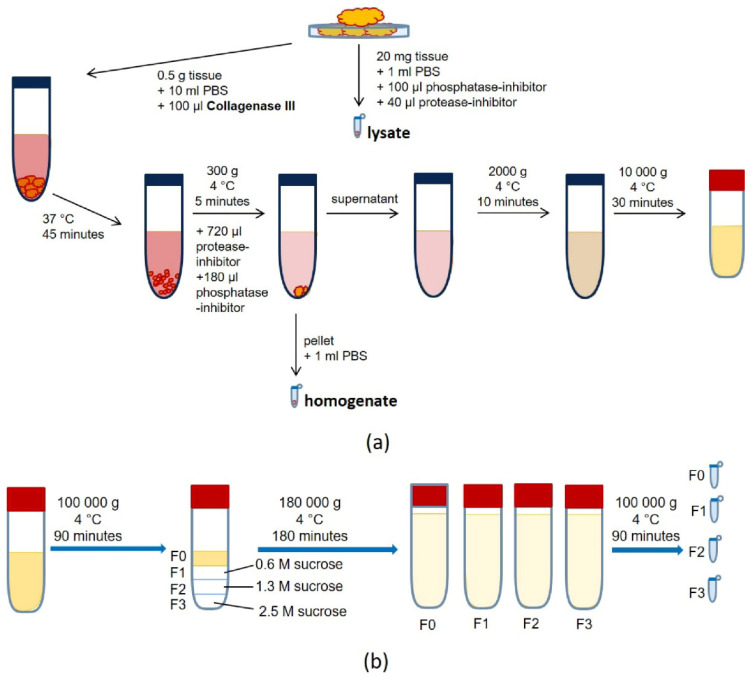
Schematic workflow for the exosome isolation from primary kidney tissue via (**a**) tissue preparation by cell dissociation (digestion with type 3 collagenase) and the removal of cell debris and larger fragments by differential centrifugation, followed by (**b**) exosome enrichment and purification by means of ultracentrifugation and a sucrose-dense gradient.

In detail, we cut approximately 500 mg of normal or tumor ccRCC tissue into small fragments. We mixed 20 mg with lysis buffer (including phosphatase and protease inhibitor) defined as tissue lysate (Figure 1). The remaining tissue was incubated in 10 mL PBS + 100 µL Collagenase Type III at 37 °C for 45 min. Subsequently, 720 µL protease inhibitor and 180 µL phosphatase inhibitor were added, and the samples were centrifuged (300 *g*/4 °C/5 min). The pellet was resuspended in 1 mL PBS (“homogenate”), and the supernatant was centrifuged again two times (2000 *g*/4 °C/20 min, 10,000 *g*/4 °C/30 min). Afterward, the supernatant was spun at 100,000 *g* at 4 °C for 90 min, and the corresponding pellet was resuspended in 6 mL PBS and defined as fraction 0 (=F0). A sucrose gradient was added to the ultracentrifuge tube (F3: 2.5 M, 2 mL; F2: 1.3 M, 2 mL; F1: 0.6 M, 2 mL), and the suspension was layered on top (F0: 6 mL). The tubes were centrifuged at 180,000 *g* at 4 °C for 180 min.

The layers (F0, F1, F2, and F3) were dissolved in PBS and centrifuged at 100,000 *g* at 4 °C for 90 min to wash the exosome pellet. The final pellet was dissolved in 50–100 µL PBS (for nano tracking analysis, NTA, or transmission electron microscopy, TEM) or lysis buffer (with protease inhibitor mix). The protein concentration was determined using a BCA assay, and the samples were stored at −20 °C in lysis buffer/protease inhibitor, or at 4 °C in PBS.

### 2.5. Characterization of Exosomes

To analyze the size and morphology of the isolated particles, the samples resuspended in PBS underwent TEM (FEI Tecnai 13, Hillsboro, OR, USA). For this purpose, the samples were fixed in 100 µL 2% PFA–PBS. A 20 µL drop of the sample was placed on parafilm for 1 min. A carbon-coated copper grid was placed on the drop, and incubated for 30 min, followed by washing with Aqua Dest (three times), and then fixed with 1% glutaraldehyde for 5 min. The grid was then washed three times with distilled water and contrasted with 1% uranyl acetate for 1 min. After 10 to 15 min, TEM was carried out.

An NTA device (NanoSight LM10, NTA Version: NTA: 3.3 Dev Build 3.3.301, Malvern Panalytical, 34123 Kassel, Germany) served to quantify and determine size. Therefore, the samples dissolved in 100 µL PBS were loaded into the measuring chamber in triplicate. The protocol was based on the manufacturer’s specifications for measurements of particles in an aqueous solution, considering the special refractive index of PBS.

Western blot was performed to semiquantitatively determine the protein expression of the cell and exosome samples. We loaded 5 to 10 µg of proteins on a polyacrylamide gel. Gel electrophoresis was run at 25 mA 200 V, for 45 min. Semi dry blotting was performed at 100 mA 200 V, 250 W at room temperature for 60 min with a polyvinylidene fluoride membrane (PVDF membrane). The PVDF membrane was blocked by incubating with a bovine serum albumin Tris-buffered saline or with a milk Tris-buffered saline with Tween20 (5% BSA–TBST or 5% milk–TBST) solution for 60 min at room temperature on a shaker. The primary Antibodies (1:1000) against exosome (CD63/ab134045/Abcam, Cambridge, UK, CD9/13174S/Cell Signaling, Danvers, MA, USA, CD81/ab109201/Abcam, Syntenin/ab133267/Abcam, Cambridge, UK), cellular (GM130/12480S/Cell Signaling, Danvers, MA, USA), and tumor markers (CA9/1:10,000/monoclonal from hybridoma/Integra, a gift from Radboud University Medical Centre Nijmegen, The Netherlands, CD147/ab108308/Abcam, Cambridge, UK, CD70/ab175389/Abcam, Cambridge, UK) were used in combination with horse reddish peroxidase coupled secondary antibodies (1:2000). Fluorescence images were taken using a Fluorescence Imager (ChemoStar electrogenerated chemiluminescence phospo-chemiluminescence, electrogenerated chemiluminescence and Fluorescence Imager, Intas Science Imaging Instruments GmbH, Göttingen, Germany) at an excitation wavelength of approximately 477 nm.

### 2.6. Immunohistochemistry

The FFPE tissue sections (4 µm) were deparaffinized (1 h, 60 °C), treated with xylene (three times per 10 min), and rehydrated within a decreasing alcohol series in five-minute steps (100%–100%–70%–70% ethanol, Aqua Dest). Antigen retrieval (10 mM citrate buffer pH 6.0 or 10 mM Tris 1 mM ethylenediaminetetraacetate (EDTA, pH 9.0)) was performed for 10–15 min at 95 °C. Afterwards, the sections were blocked with BSA (3% BSA in PBS (w/v), pH 7.2) for 30 min, washed with PBS, and blocked with biotin (Biotin Block from DCS Innovative Diagnostik-Systeme, Dr. Christian Sartori GmbH & Co. KG, 22397 Hamburg, Germany) for 20 min, followed by washing with PBS (20 min). The tissue sections were incubated with primary antibodies (solved in 1% BSA in PBS, pH 7.2) against CD70 (monoclonal mouse, MAB2738, RD Systems), CD147 (monoclonal rabbit, ab108308, Abcam), CA9 (recombinant mouse, monoclonal from hybridoma, Integra), or EpCAM (monoclonal rabbit, D4K8R, Cell Signaling) for 1 h at 37 °C and then washed four times with PBS. Detection was performed with the REAL Detection System Alkaline Phosphatase/RED chromogene/ rabbit/mouse (Dako/Agilent, Santa Clara, CA, USA) according to the manufacturer′s protocol.

## 3. Results

### 3.1. Exosome Isolation from Cell Culture

**Figure 2:**
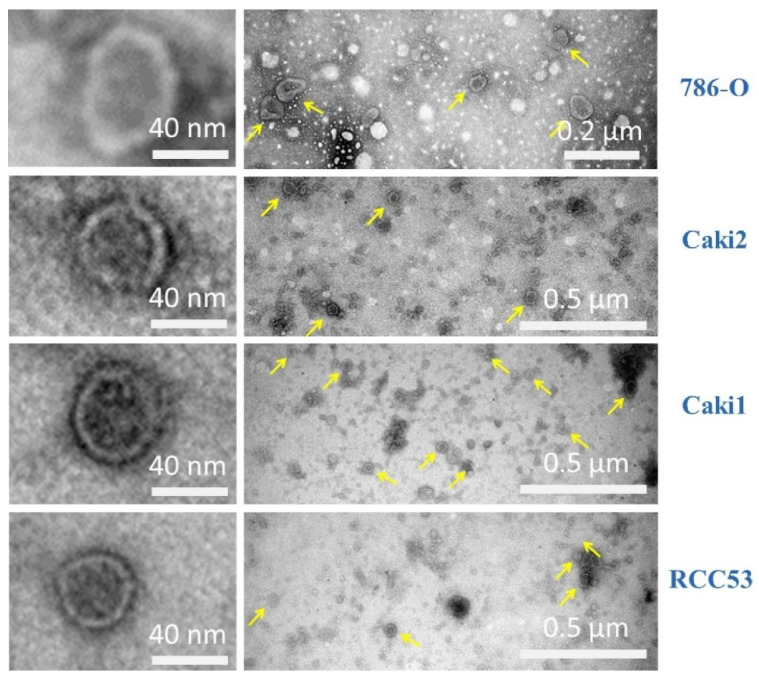
Exosomes detected by transmission electron microscopy (TEM) in cell lines. Left panel: Magnification of single exosomes. Right panel: Widefield.

**Figure 3:**
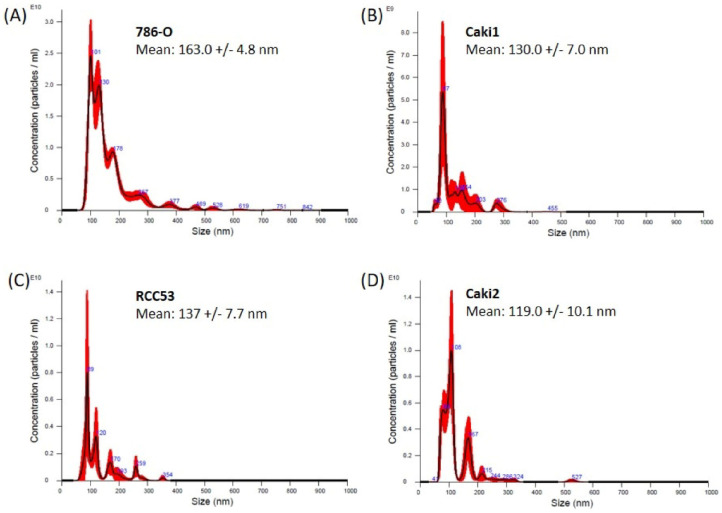
Nanoparticle tracking analysis (NTA) of exosomes derived from human clear cell renal carcinoma (ccRCC) cell lines (**A**) 786-O, (**B**) Caki1, (**C**) RCC53, and (**D**) Caki2 cell culture supernatants. The red bars indicate the standard error of the mean values from the measurements (*n* = 3).

**Figure 4:**
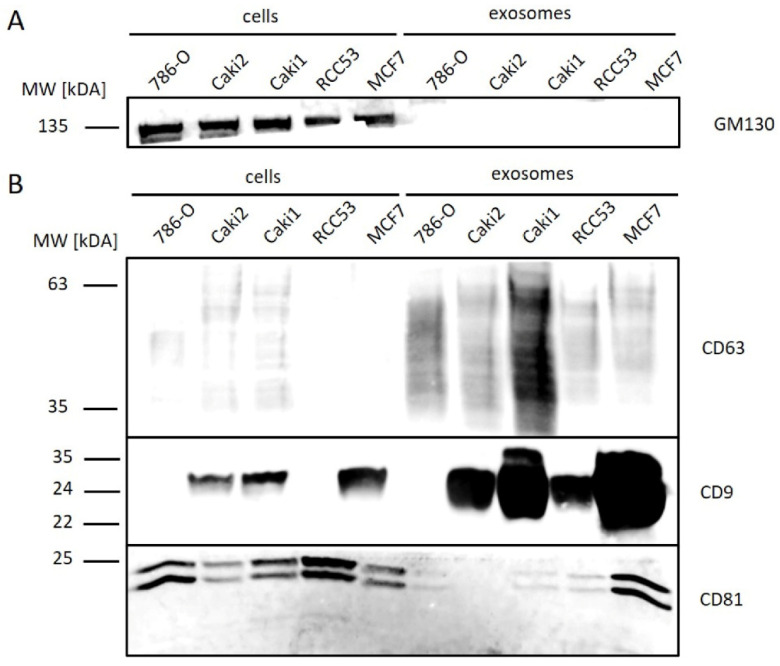
Western blot analysis of exosomal and cellular proteins from ccRCC (786-O, Caki2, Caki1, and RCC53) and control (MCF7) cell lines. (**A**) cell marker, (**B**) exosome marker.

We successfully isolated exosomes from cell culture supernatants with adequate purity and concentration (Figure 2, Figure 3 and Figure 4). Transmission electron microscopy (TEM) images showed spherical particles with double membranes and a size between 45 and 53 nm in all four cell lines (Figure 2).

Nanoparticle tracking analysis (NTA) revealed a size distribution with a peak at approximately 100 nm in all four tested ccRCC cell lines, reflecting the size distribution of exosomes (Figure 3A–D).

To confirm the quality of exosome isolation, we analyzed the expression of cellular (Golgi marker 130 (GM130)) and exosomal (cluster of differentiation 63, 81, 9 (CD63, CD81, and CD9)) proteins via Western blot (Figure 4). GM130 was detectable in all cells, but not in the corresponding exosome samples, underlining a high purification of exosomes without cellular contaminants (Figure 4A). CD63 was enriched in all exosome samples, with the highest concentration found in Caki1. CD9 was detected in all exosome samples, except for 786-O, with higher signal intensities in all exosome samples than in the corresponding cell samples. CD81 was observed at low concentrations in exosome samples of ccRCC cell lines in contrast to MCF7 (Michigan Cancer Foundation—7)-derived exosomes, which served as a control (Figure 4B).

### 3.2. Exosome Isolation from Tissue Samples

**Figure 5:**
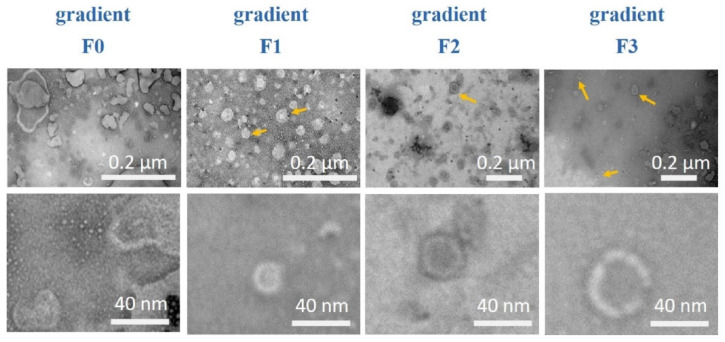
Exosomes from tumor tissues in different gradient fractions detected via TEM. Upper panel: Widefield, exosomes from tumor tissue. Lower panels: magnification of a single exosome from tumor tissue.

**Figure 6:**
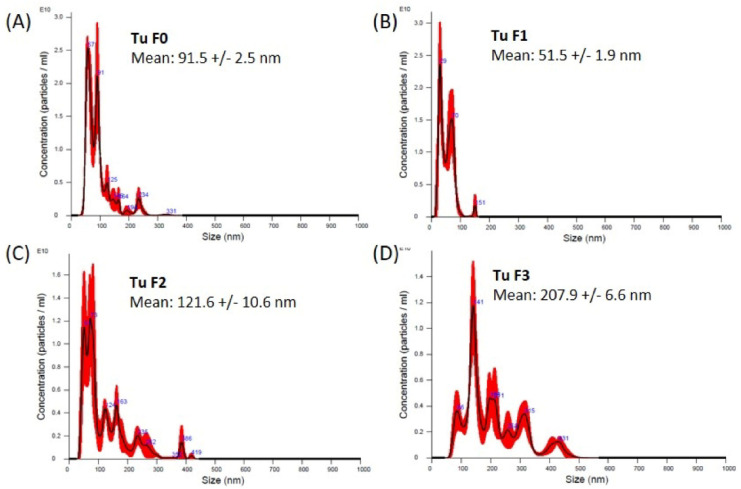
Nano tracking analysis of exosomes from primary tumor tissue sample isolated by sucrose gradient. The size distribution of the fractions F0–F3 (**A**–**D**) is shown as the average of three measurements. The red error bars indicate the standard error of the mean values from three measurements. Tu = tumor.

In order to isolate exosomes directly from human tissue samples, we established a specific protocol combining a sucrose density gradient with ultracentrifugation (Figure 1). TEM images confirmed spherical particle shapes with double membranes and a size between 36 and 120 nm in all ccRCC tumor tissue samples (Figure 5, lower panel). The impurities decreased with increasing density of the gradient; thus, the purity of the isolated exosomes increased (Figure 5, upper panel). NTA revealed a size between 50 and 210 nm in exosomes from tumor tissue samples (Figure 6A–D).

**Figure 7:**
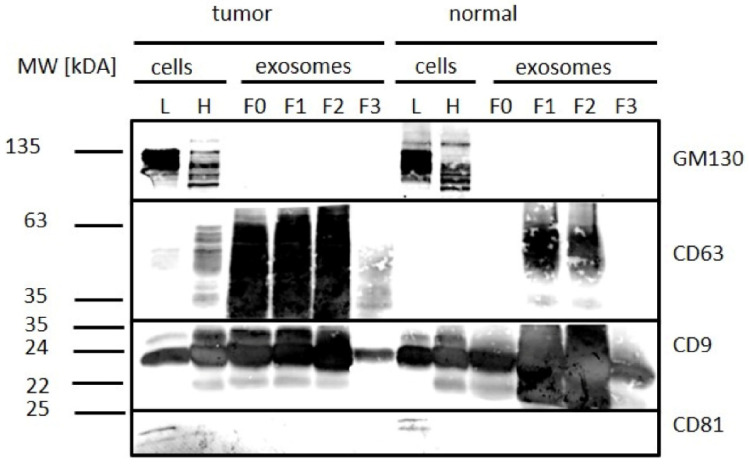
Western blot analysis of exosomal and cellular proteins, isolated from tumor and normal tissue. Cell (Golgi marker 130 (GM130)) as well as exosome markers (CD63, CD9, and CD81) were detected. L = lysate, H = homogenate, F0–F3 = fraction F0–F3 (fractions after sucrose gradient).

GM130 was detectable in all tissue samples (lysate and homogenate), but not in their corresponding exosome samples, by Western blot (Figure 7). CD63 was detectable in all exosomal fractions of tumor samples, and the signal intensity was reduced in fraction 3 (F3), whereas it was detected only in F1 and F2 from normal tissue samples. CD9 was enriched in fractions 0, 1, and 2 and decreased in fraction 3 in both normal and tumor tissue exosomes. CD81 was not observed in exosome samples. In addition to the three tetraspanins, we tested Syntenin, which is a vesicle-associated protein. Syntenin was found to be increased in the exosomal fractions (Figure S1).

### 3.3. Expression of Tumor Markers in Cells and Tissues

#### 3.3.1. Cell Culture

**Figure 8:**
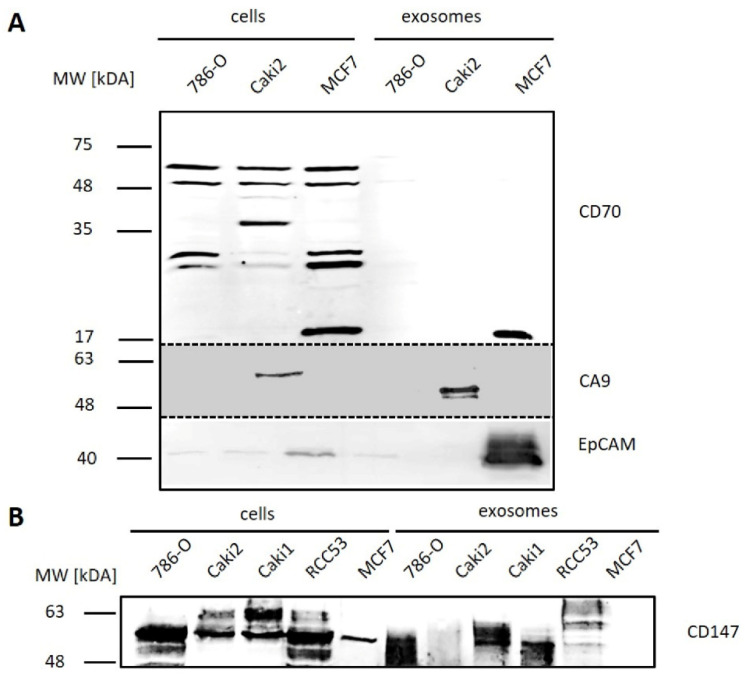
Western blot analysis of tumor-associated markers. (**A**) Carbonic anhydrase 9 (CA9), CD70, and epithelial cell adhesion molecule (EpCAM); (**B**) CD147) on exosomes from ccRCC (786-O, Caki2, Caki1, and RCC53) and control (MCF7) cell lines.

CD147 and CA9 were detected in cells and exosomes from ccRCC cell lines with cell-line specific expression patterns (Figure 8). CD70 occurred only in the MCF7 control cells and their exosomes as a specific band at 17 kDA.

EpCAM, selected as a standard epithelial tumor cell marker, was found to be weakly or not expressed in cells and exosomes from ccRCC cell lines in contrast to the control cell line MCF7 (Figure 8). The expression of CA9 was observed in Caki2 cells and their exosomes, but not in 786-O cells or exosomes. CA9 signals in exosomes showed a double band at 50 and 55 kDa, and a single band in cell samples of Caki2. CD70 was not detected at the expected size of 21 kDA, but at higher size (30/32 kDa, 38 kDa, 46 kDa, 50 kDa, and 60 kDa). As the most promising marker, CD147 was tested in four ccRCC cell lines. It was expressed with equal or higher concentrations on exosomes of 786-O, Caki1, and RCC53 cell lines and with decreased concentrations on exosomes of Caki2 compared to the corresponding parental cells.

#### 3.3.2. Primary Tumor Tissue

**Figure 9:**
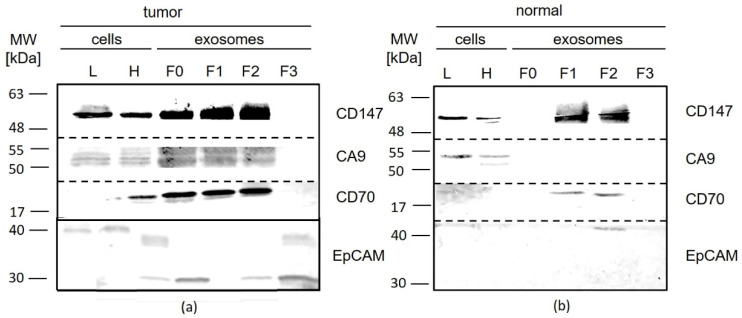
Western blot analysis of tumor-associated markers (CD147, CA9, CD70, EpCAM) in samples derived from renal tumors and normal tissues (NTB1175 and NTB1302). L = lysate, H = homogenate, F0–F3 = fractions 0–3, MW = molecular weight, kDa.

In primary tumor tissues, EpCAM was found to be expressed in 5/8 cellular and EV fractions, with increased concentrations of cleaved fragments in all four fractions. In normal tissue, it was not detected in exosome fraction 2 (Figure 9, Table 1 and Table 2, Supplementary Table S1).

CA9 was detected in all primary tumor tissues and their exosomes. Fractions 0–2 revealed increased signal intensities compared to the cellular samples (Figure 9, L, H), and fraction 3 lacked any signal for CA9. In contrast, CA9 was detected in the cellular samples, and it was found to be weakly expressed in the exosome fractions from normal tissues (Figure 9, Table 1 and Table 2, Supplementary Table S1).

CD70 exhibited different expression patterns between tumor tissues (8/8) and their EVs (5/8), depending on the patient sample. Fractions 0–2 showed increased signal intensities compared to the cellular samples (Figure 9, L, H). CD70 expression was increased in tumor samples compared to normal tissue. It was detected in F1 and F2, but not in F0 and F3, and it was at a very low level in the cell lysates from normal tissue (Figure 9, Table 1 and Table 2, Supplementary Table S1).

CD147 was strongly expressed in the cell lysates and exosomes in all primary tumor tissue samples (Figure 9, Table 1). Exosome fractions 0–2 had increased signal intensities compared to the corresponding cells (Figure 9, L, H). The expression was increased in tumor samples in comparison with normal tissues (Figure 9, Table 1 and Table 2, Supplementary Table S1).

#### 3.3.3. Immunohistochemistry

**Figure 10:**
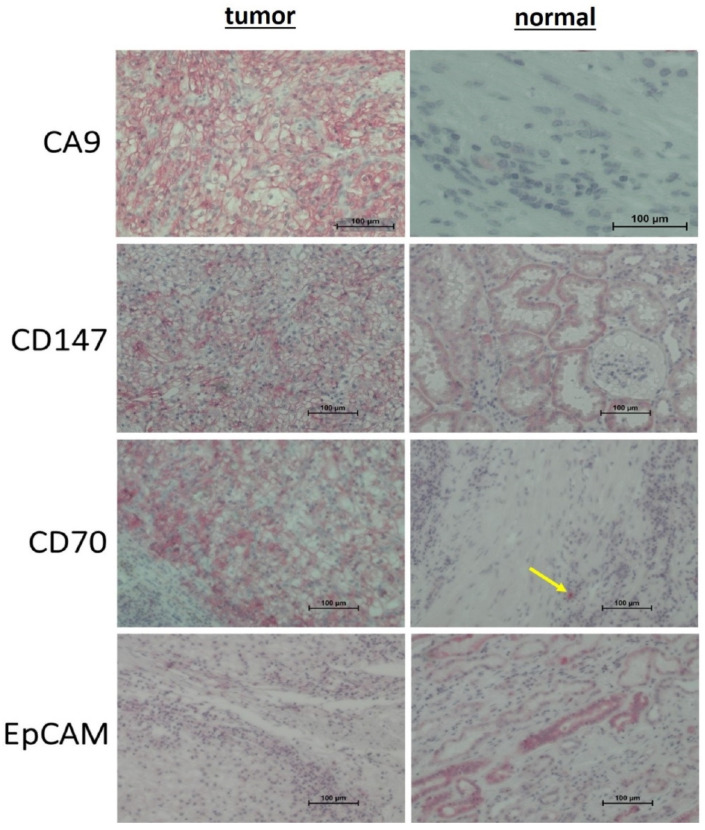
Representative images of the immunohistochemical staining of tumor markers (CA9, CD147, CD70, and EpCAM) on formalin-fixed paraffin-embedded (FFPE) sections of tumor and normal primary tissue samples from patients with clear cell renal carcinoma (ccRCC). Yellow arrow indicates an infiltrating immune cell. Scale bar = 100 µm.

To identify the cellular origin of the analyzed marker proteins, immunohistochemistry (IHC) was performed on a larger series (*n* = 13) including the corresponding tissue sections analyzed by Western blotting (Figure 10).

CA9 was highly expressed in tumor cells in all cases and was moderately expressed in normal tissue, confirming the Western blot results. The tumor cells showed a continuous intensive staining of CA9. In normal cells, expression was found in 11 out of 13 cases by IHC predominantly with low or moderate expression (Tables S1 and S2).

CD147 was expressed in normal tubular and in tumor cells with higher signal intensity in tumor cells. Both tumor and normal tissue showed homogeneous staining. In the tumor cells, CD147 was detected in all cases by IHC (moderate staining), as also shown by Western blot. In normal cells, we found expression in 8 of 13 by IHC, predominantly with weak expression, confirming the Western blot results (Tables S1 and S2).

CD70 was highly expressed in all cases in tumor cells but not in normal tissue (Tables S1 and S2). Infiltrating immune cells in normal tissues displayed a strong CD70 signal (Figure 10, yellow arrow).

EpCAM was expressed in normal tubular and tumor cells at a similar, heterogenous expression level. Some samples had a very weak or no signal within tumors in contrast to adjacent normal tissue with a very pronounced expression in some cases Table S1). In the tumor cells, moderate or strong expression of EpCAM was detected in 11 of 13 cases by IHC. We found that 12 out of 13 normal tissues were positively stained with different intensities (Tables S1 and S2).

## 4. Discussion

The aim of this study was to identify tumor-specific proteins on exosomes as potential biomarkers for the targeted enrichment of tumor-specific exosomes from liquid biopsies. The isolation of exosomes represents a major challenge due to their small size and the high risk of contamination with cellular fragments and lipoprotein complexes. In addition, we considered it important to distinguish exosomes from other vesicles arising by apoptosis or membrane budding. To optimize exosome isolation, we established a standardized protocol for ultracentrifugation on four different ccRCC cell lines. We found cell line-dependent size distributions (as detected by NTA) and the typical protein expression patterns of exosome markers.

The differences regarding the measured sizes of the exosomes in TEM and NTA presumably resulted from the more effective bundling of small exosomes on the coal-copper grid that was used for the TEM. Therefore, NTA is the preferred method for determining the size distribution of exosomes in solution. As CD9 was not expressed in exosomes throughout all ccRCC cell lines, it cannot serve as a general marker for exosomes from tumor cells. CD81 did not represent a suitable exosome marker for ccRCCs in cell culture either, as its expression was highly different between cell lines and absent in exosomes. However, we found a stable expression of CD63, confirming its value as a universal exosome marker according to the current guidelines of exosome isolation by the International Society of Extracellular Vesicles [44].

As the cell lines differ in morphology and metabolism, it is likely that their exosomes also differ in size, number, and surface markers. This heterogeneity was also reflected in the expression of tumor markers. We selected three putative tumor markers and EpCAM as an epithelial cell marker. CD70 was detected only as multiple bands (multiples of the expected molecular weight), apparently due to the formation of multimers in the cell samples, and this did not occur on 786-O and Caki2 exosomes. CA9 was expressed only in the Caki2 cell line and was further enriched in their exosomes. Only CD147 showed a strong expression in both 786-O and Caki2 cells. It was, therefore, tested on two other ccRCC cell lines (Caki1 and RCC53). We confirmed that CD147 was prominently expressed in all ccRCC cell lines and their exosomes. The molecular weight varied between the cell lines, which can be attributed to different glycosylation patterns [45]. Apart from the control cell line MCF7, the expression of EpCAM was very low or below the detection limits.

Although established cell lines represent effective models for in vitro studies, these models often significantly differ from in vivo situations due to tumor clone selection and missing communication within the tumor microenvironment. Therefore, we further focused our investigations on primary tumor tissues from patients. Due to the much stronger cell–cell contacts, the extraction and purification of exosomes from tissues poses a great challenge. To date, there are only few data on the isolation of exosomes from kidney tissue. Zieren et al. reported the isolation of exosomes based on collagenase treatment, differential centrifugation, and filtration with 800 nm and 450 nm filters, followed by ultracentrifugation [46]. In contrast, our protocol was based on a density gradient without mechanical filters. To the best of our knowledge, this is the first report on successful exosome isolation from tumor and normal kidney tissues samples based on ultracentrifugation combined with a sucrose gradient. By applying a sucrose gradient to deplete the contaminants present in kidney tissues, we successfully isolated the corresponding exosomes at a high concentration and purity, as confirmed by Western blot, TEM, and NTA. Thereby, we found 1.3 M sucrose to be the optimal concentration. The comparison with exosomes from cell culture revealed differences between the expressions of the tumor-specific marker proteins.

Verification of the isolated exosomes was based on the current quality standards and guidelines on the characterization of extracellular vesicles [44]. In addition to the size, the source of exosomes plays an important role in the selection of the isolation method. Our isolation protocol was optimized in order to obtain the best possible ratio of purity and concentration while being cost- and time-efficient [46,47,48].

The expression of tumor-specific markers on exosomes reflects the cellular origin of the EVs [49]. To confirm that the proteins found by Western blot originated from the tumor cells, we performed immunohistochemical staining. EpCAM, a cell adhesion molecule, is known to be moderately expressed in normal epithelia, and to be overexpressed in certain tumor types, including RCC [50]. EpCAM has, moreover, been associated with the initiation of diverse tumor types, and also with disease recurrence and poor clinical outcomes [51,52]. However, the expression of EpCAM significantly varies in normal kidney tissue [53], and it ranged from moderate to strong in our tumor samples, but was enriched in their exosomes. Our data show that EpCAM expression in kidney tumor tissues was principally not stronger than in normal tissue.

After digestion and centrifugation, only fission products were detected from EpCAM in the individual fractions of the sucrose gradient. These alterations have already been described in previous studies [54]. Protein cleavage frequently occurs in tumors due to dysregulation during tumorigenesis [55,56]. Likely, the intact exodomain of EpCAM is recovered in a soluble form and enriched in the exosome fraction in RCC. However, EpCAM expression decreases during cellular dedifferentiation. Correspondingly, a recent study proved that the expression of EpCAM decreased in more aggressive RCC as well [57]. As EpCAM is weakly expressed on exosomes and heterogeneously expressed in both tumors and normal tissues, it is not suitable for tumor detection using exosomes.

To overcome these limitations of EpCAM, we decided to test CD147, CA9, and CD70 as potential tumor markers for ccRCC. CD147 has already been well described as a tumor-specific protein in various tumor entities [58,59,60,61,62,63,64,65]. This transmembrane glycoprotein is highly homologous to proteins of the immunoglobulin (Ig) superfamily. CD147 is involved in matrix degeneration, tumor cell invasion, metastasis, and angiogenesis via the regulation of glycosylation and the induction of proteinases [58]. Both CD147 and its ligand MMP-9 (matrix metalloprotease-9) are overexpressed in RCC [59,60]. CD147 upregulation in tumor cells is associated with poor prognoses [61,62]. In addition, CD147 is overexpressed in patients treated with sunitinib therapy and in sunitinib-resistant 786-O cells [63]. In line with other studies [64,65], our data suggest that parts of normal tissues express CD147 as well. However, the expression was strong in the tumor-associated exosomes, supporting its role as a putative marker for RCC-released exosomes. The expression of CD147 in normal cell exosomes has to be further evaluated.

CA9 is well characterized in ccRCC [66]. The strong overexpression of CA9 in our tumor samples confirmed the results of previous studies [66]. Carboanhydrases can facilitate renal acidification, as the concentrations of CO_2_ and HCO_3_ are interdependent. CA2 and CA4 occur in healthy human kidneys, whereas CA9 is only present in tumor tissue [67,68,69]. Although we found a weak expression in the tubuli of normal tissue in certain cases by Western blot and immunohistochemistry, CA9 was found to be weakly expressed on the exosomes from normal cells. In contrast, tumor cells showed a strong expression of CA9, which was further increased on the tumor exosomes. IHC confirmed the strong expression in the ccRCC tumor samples. The results from Mulders et al. showed that this marker could already be used as a diagnostic and therapeutic target [70].

CD70 was found to be expressed in ccRCC, too [71]. CD70 represents a ligand of CD27 and plays an important role in the generation and maintenance of T-cell immunity. Upon CD27 binding, CD70 induces the proliferation of co-stimulated T-cells and triggers the generation of cytolytic T-cells [72,73]. In addition to its physiological function, CD70 also appears to interact with tumorigenesis. The constitutive expression of CD70 has been described in various solid tumor types, particularly in ccRCC [74]. Within our study, we clearly confirmed the diagnostic potential of CD70. The weak expression of CD70 in normal tissue by Western blot was not confirmed by IHC, indicating that normal epithelial cells in the kidney did not express CD70. Infiltrating immune cells and their exosomes most likely express CD70, resulting in weak positive signals in cell lysates and exosomes as demonstrated by Western blotting. The differences between cell culture and tumor tissue samples can be explained by the lack of contact with the microenvironment, including immune cells that might be necessary for the induction of CD70 expression. To conclude, CD70 was strongly expressed in ccRCC tumor tissue and could serve as a ccRCC-specific exosome marker.

Our findings demonstrate that cell lines represent suitable models for characterizing exosomes in vitro. However, the specific characteristics of tumor cells and their exosomes can be missed due to the lack of cell–cell communication with the tumor microenvironment.

Our study has some limitations. The amount of tissue sample material was limited, in particular regarding normal tissue samples. Therefore, not all markers could be examined in all samples and in comparison with the adjacent normal counterpart. However, this study represents the largest series to investigate exosomes from primary ccRCC tissues to date. Future studies with larger patient cohorts will increase the reliability of our findings.

## 5. Conclusions

We developed an effective technique to isolate exosomes directly from human renal tissue samples with high purity and high concentration. The expression of tumor-specific markers reflects the cellular background of EVs. In contrast to EpCAM, CA9, CD70, and CD147 represent promising tumor-specific biomarkers for EVs in ccRCC. Based on these results, further investigations will focus on the development of techniques to enrich tumor-specific EVs from body fluids using these promising markers.

## Figures and Tables

**Table 1:** The detection of tumor-associated markers (CD147, CA9, CD70, and EpCAM) by Western blot in cells and tumor-derived exosomes from patient samples. The number of positive samples is related to the total sample number analyzed.

Origin	CA9	CD147	CD70	EpCAM
cells	6/6	8/8	8/8	5/7
exosomes	7/7	8/8	5/8	5/8

**Table 2:** Histopathological characteristics and Western blot results on tumor tissue samples. Semiquantitative evaluation: “-” = no expression; “+” = low expression; “++” = moderate expression; “+++” = high expression; n.e. = not examined. TNM = Tumor Node Metastasis Classification of Malignant Tumors.

Case No.	Cells				Exosomes				TNM	Grade
	CA9	CD147	CD70	EpCAM	CA9	CD147	CD70	EpCAM		
NTB1126	n.e.	+	+	n.e.	+++	+	-	-	pT3acN0cM0	unknown
NTB1302	+++	++	+	n.e.	+++	+++	-	n.e.	pT3acN0cM0	G3
NTB1161	n.e.	+	+	n.e.	n.e.	+	-	n.e.	pT1bcN0cM0	G2
NTB1208	+	+	+	+	++	+	++	+	pT4cN0cM1	G3
NTB1175	++	+	+	-	+++	++	++	-	pT3acN0cM0	G3
NTB1288	++	+	+	-	++	+	+++	-	pT3acN0M0	G3
NTB1304	n.e.	n.e.	n.e.	++	n.e.	n.e.	n.e.	++	pT3bpN0M0	G2
NTB1132	n.e.	n.e.	n.e.	+	n.e.	n.e.	n.e.	+++	pT1bcN0cM0	G2
NTB1197	++	+	+	+	+++	+++	+++	++	pT1bcN0cM0	G2
NTB1272	+++	+	+	++	+++	++	++	+	pT1bcN0cM0	G2

## Supplementary Materials

The following are available online at https://www.mdpi.com/2075-4418/10/12/1034/s1.
