# Supplementary material for: Characterization of CD147, CA9, and CD70 as Tumor-Specific Markers on Extracellular Vesicles in Clear Cell Renal Cell Carcinoma
Source: Diagnostics (Basel). 2020 Dec 2;10(12):1034. doi: 10.3390/diagnostics10121034 (PMC7761541; doi:10.3390/diagnostics10121034)
Supplement: Supplementary file 1 [file diagnostics-10-01034-s001.pdf]

Table S1: Quantification and comparison of expression patterns in normal tissue samples. “-“ = no expression; “+” = low expression; “++” = moderate expression; “+++” = high expression; n.e. = not examined.

	cells				exosomes			
**Case no.**	CA9	CD147	CD70	EpCAM	CA9	CD147	CD70	EpCAM
NTB1126	n.e.	+	+	n.e.	+	-	-	-
NTB1302	++	+	-	n.e.	+	+++	-	n.e.
NTB1161	n.e.	+	+	n.e.	-	-	-	-
NTB1175	+	+	+	-	+	++	+	-

Table S2: Quantification and comparison of expression patterns in tissue samples. Weighting: “-“ = no expression; “+” = low expression; “++” = moderate expression; “+++” = high expression.

	EpCAM				CD147				CA9				CD70			
Case	tumor		tubuli		tumor		tubuli		tumor		tubuli		tumor		tubuli	
NTB	%	Intensity	%	Intensity	%	Intensity	%	Intensity	%	Intensity	%	Intensity	%	Intensity	%	Intensity
1194	0	0	40	++	10	+	10	++	100	+++	80	++	40	+	0	0
1132	60	++	0	0	20	++	0	0	100	+++	0	0	70	++	0	0
1160	70	++	100	++	30	++	30	+	100	+++	40	0	70	++	0	0
1304	0	0	50	+	70	++	0	0	100	+++	20	+	90	++	0	0
1233	60	++	80	++	10	++	70	++	100	+++	50	+	0	0	0	0
1125	70	++	100	+++	20	++	0	0	100	+++	0	0	80	+	0	0
1288	30	++	90	+++	30	++	30	++	100	+++	60	++	70	++	0	0
1161	40	++	70	++	20	++	20	+	90	+++	20	+	20	+	0	0
1302	50	++	80	++	80	++	20	++	100	+++	30	+	90	++	0	0
1272	30	++	30	++	60	++	0	0	100	+++	40	++	70	++	0	0
1208	20	++	20	++	60	++	20	+	40	++	20	+	0	0	0	0
1197	10	+	40	++	20	++	0	0	80	++	10	+	70	++	0	0
1175	20	++	80	++	90	+++	10	+	100	+++	40	+	80	+++	0	0

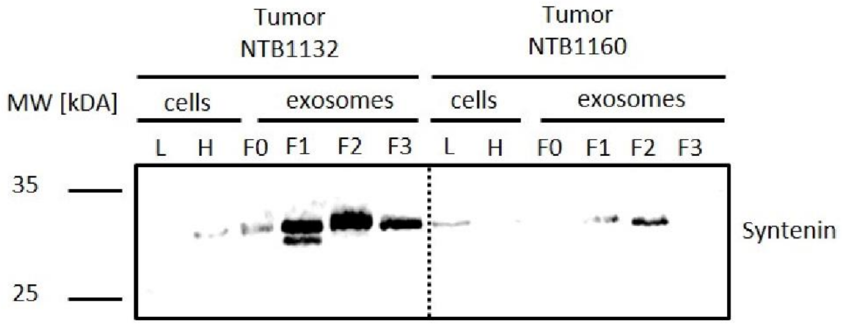

Figure S1: Western blot analysis of the exosomal marker Syntenin, isolated from two representative tumor tissues. L = lysate, H = homogenate, F0–F3 = fraction F0–F3 (fractions after sucrose gradient)
